# Identification of cellular senescence-related signature for predicting prognosis and therapeutic response of acute myeloid leukemia

**DOI:** 10.18632/aging.205123

**Published:** 2023-10-13

**Authors:** Fangmin Zhong, Yulin Yang, Fangyi Yao, Jing Liu, Xiajing Yu, Xin-Lu Wang, Bo Huang, Xiao-Zhong Wang

**Affiliations:** 1 Department of Clinical Laboratory, Jiangxi Province Key Laboratory of Laboratory Medicine, The Second Affiliated Hospital of Nanchang University, Nanchang, Jiangxi, China; 2 School of Public Health, Nanchang University, Nanchang, Jiangxi, China

**Keywords:** acute myeloid leukemia, cellular senescence, tumor microenvironment, prognosis, therapeutic response

## Abstract

Cellular senescence is closely related to the occurrence, development, and immune regulation of cancer. However, the predictive value of cellular senescence-related signature in clinical outcome and treatment response in acute myeloid leukemia (AML) remains unexplored. By analyzing the expression profile of cellular senescence-related genes (CSRGs) in AML samples in the TCGA database, we found that cellular senescence is closely related to the prognosis and tumor microenvironment of AML patients, and compared with normal samples, the overall expression level of senescent inducing genes in AML samples was down-regulated, while inhibitory genes were up-regulated. The risk score model further constructed and verified based on CSRGs could be used as an independent prognostic predictor for AML patients, and the overall survival (OS) of high-risk patients was significantly shortened. The area under ROC curve (AUC) values for the prediction of 1-, 3- and 5-year OS were 0.759, 0.749, and 0.806, respectively. In addition, patients with high-risk scores are more sensitive to treatment with cytarabine and may benefit from anti-PD-1 immunotherapy. In conclusion, our results suggest that the cellular senescence-related signature is a strong biomarker of immunotherapy response and prognosis in AML.

## INTRODUCTION

Acute myeloid leukemia is highly malignant and patients have a poor prognosis [1]. With current advances in treatment, many patients have significantly longer survival times, but remain incurable [2]. Therefore, it is important to explore new therapeutic targets and prognostic predictors.

Many studies have shown that individual aging influences cellular senescence, and the biological processes associated with cellular senescence are closely related to a variety of diseases including cancer [3, 4]. As a stress response, cellular senescence is characterized at the genomic level by various degrees of exhaustion and impaired signaling functions [3, 5, 6] and induces gene expression variation [7]. Moreover, cellular senescence is closely associated with the tumor microenvironment (TME), including the activation of various cancer-promoting signaling molecules and cytokines [8–10], and plays a role in promoting the accumulation of multiple immunosuppressive cells. These biological behaviors profoundly influence the remodeling of the TME and tumor survival [11, 12]. More importantly, malignant behavior mediated by cell senescence can disrupt the adaptability of immune cells, thus inhibiting the effect of anti-tumor immunity and related immunotherapy [13, 14]. However, how cellular senescence affects the TME remains unclear, and the value of cellular senescence-related genes (CSRGs) in evaluating patient prognosis and therapeutic efficacy needs to be further explored.

In this project, based on the transcriptomic data of AML samples, we analyzed the relationship between CSRGs and the activity changes of signaling pathways and immune cell infiltration characteristics. We also constructed and validated a CSRG-based scoring model to predict overall survival (OS) and therapeutic response to chemotherapeutic drugs or immune checkpoint inhibitors (ICIs) in individual AML patients. These bioinformatics results reveal a link between CSRGs and the TME in AML and assist in the assessment of AML patient survival and treatment.

## METHODS

### Data acquisition and processing

We downloaded the normalized RNA-seq data (TPM values) of 173 TCGA-LAML (The Cancer Genome Atlas-Acute Myeloid Leukemia) samples and 337 normal GTEx (Genome Tissue Expression)-whole blood samples from the UCSC XENA database (https://xenabrowser.net/datapages/). For the validation cohorts GSE14468 and GSE10358, we downloaded the original microarray data “cel” file from Gene Expression Omnibus (GEO) database (https://www.ncbi.nlm.nih.gov/geo/) and used the robust multiarray averaging (RMA) method to standardize it. For another validation cohort GSE71014, we downloaded the normalized matrix file. Somatic mutation data of AML patients were downloaded from the TCGA database (https://portal.gdc.cancer.gov/). We extracted 274 CSRGs from the CellAge database (https://genomics.senescence.info/cells/), including 153 induced genes and 121 inhibited genes. Based on the expression of two types of genes, we used the gene set variation analysis (GSVA) algorithm to calculate the cell senescence induction and inhibition scores of AML patients in the TCGA database, respectively. Cell senescence score (CS-score) = induction score − inhibition score.

### Pathway activity assessment and function enrichment analysis

We used the GSVA algorithm to calculate an enrichment score for a gene set based on the expression levels of all genes in the gene set, thereby quantifying the activity of the corresponding biological process or signaling pathway. For the identification of signaling pathways with differential activity between two groups, we used the gene set enrichment analysis (GSEA) algorithm to determine. All of these were performed in the “clusterProfiler” package [15].

### Evaluation of immune cell infiltration levels

We used the CIBERSORT algorithm, which is based on support vector regression, to calculate the infiltration proportion of immune cells in AML samples [16]. The matrix file of LM22 signatures was downloaded in the supplemental file of the study.

### Construction of risk score model

We first identified genes significantly associated with prognosis in AML patients by univariate Cox regression analysis (*P* < 0.001) and constructed a risk score model by multivariate Cox regression analysis. The risk score was calculated using the following equation: Risk score = ABI3 × 0.158767529566805 + SOCS1 × 0.339547286870344 + PIM1 × 0.230120859832003 + SFN × 0.876243162175369, where gene ID refers to the expression value of each gene.

### Prediction of immunotherapy response and drug sensitivity

The half-maximal inhibitory concentration (IC50) value of each AML sample to the commonly used chemotherapy drugs cytarabine, doxorubicin, or the targeted drug midostaurin was quantified using the “pRRophetic” package based on the Genomics of Drug Sensitivity in Cancer (GDSC; https://www.cancerrxgene.org/) database. For predicting the immunotherapeutic response to ICI anti-PD-1 and anti-CTLA4 in low- and high-risk score groups, we used SubMap (https://cloud.genepattern.org/gp) algorithm to perform it.

### Real-time quantitative PCR (RT-PCR)

This project was approved by the Ethics Committee of the Second Affiliated Hospital of Nanchang University, and 15 AML samples and 15 normal samples were collected. All subjects signed an informed consent form. We extracted RNA from mononuclear cells and reverse transcribed it. The expression of the four model genes was detected by RT-PCR using the Japanese TAKARA kit on an ABI7500 detection instrument. The results were calculated using the 2^−ΔΔCT^ method.

### Statistical analysis

The Wilcoxon rank sum test and Kruskal-Wallis test were used to determine differences between two or more groups. The “survminer” package divides patients into high- and low-risk score groups based on the cut-off point at the minimum *p*-value of the log-rank test. In the Kaplan-Meier survival curve analysis, the log-rank test was used to determine the *p*-value between groups. Univariate and multivariate Cox regression analysis was used to determine the prognostic value of variables. The prediction efficiency was further verified by the analysis of the receiver operating characteristic (ROC) curve. The “maftools” package was used to demonstrate somatic mutation signatures of AML patients. A two-sided *P* value of < 0.05 was considered statistically significant.

### Availability of data and materials

All data used in this work can be acquired from the Gene-Expression Omnibus (GEO; https://www.ncbi.nlm.nih.gov/geo/) and The Cancer Genome Atlas (TCGA) database (https://portal.gdc.cancer.gov/).

### Code availability

Analyses were conducted using R (version 4.1.2). The codes used to support the findings of this study are available from the corresponding author on reasonable request.

## RESULTS

### Cell senescence is associated with the prognosis and biological characteristics of AML patients

**Figure 1:**
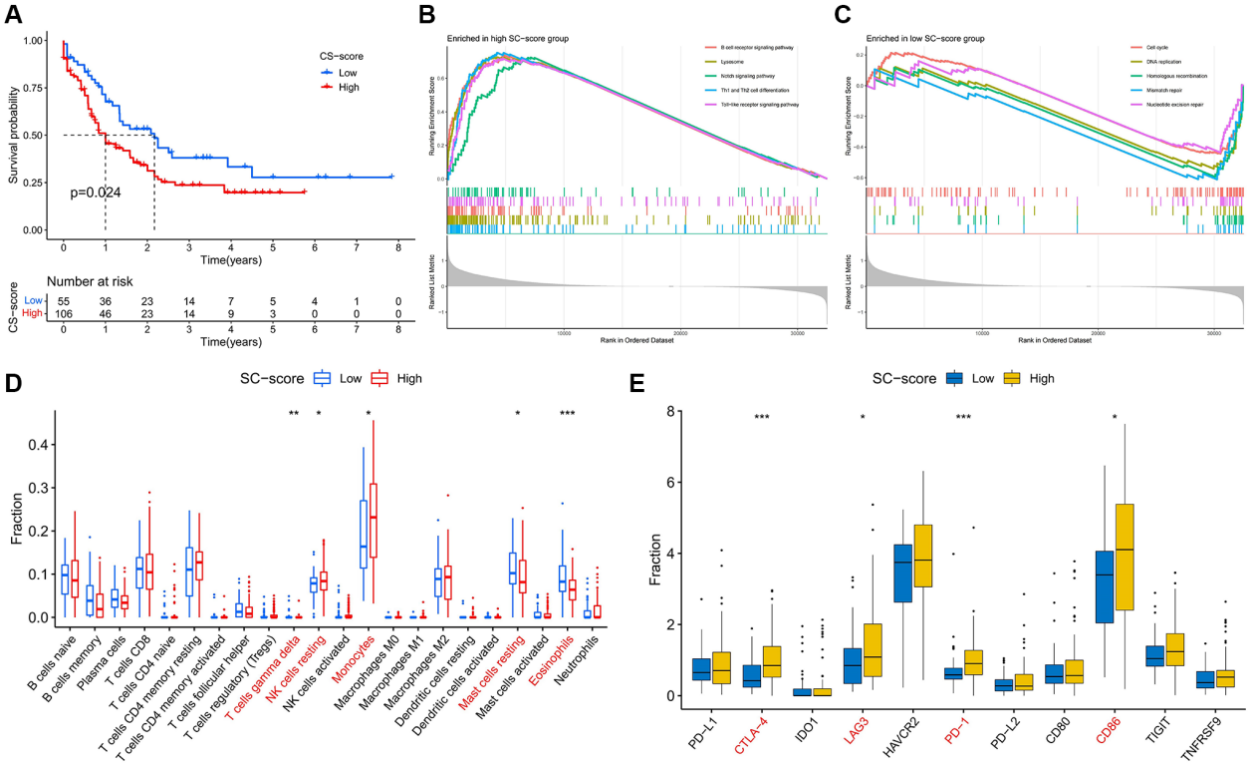
**Changes in prognosis, signaling pathways, and immune function between high and low CS-score groups.** (**A**) Differences in patients’ overall survival (OS) in the high and low CS-score groups, log-rank test. (**B**, **C**) Gene set enrichment analysis revealed the signaling pathways that were significantly enriched differently between high and low CS-score groups. (**B**) High CS-score group; (**C**) Low CS-score group. (**D**) Differences in Infiltration levels of 22 immune cells between high and low CS-score groups. (**E**) Differences in expression of immune checkpoints between high and low CS-score groups. ^*^P < 0.05, ^**^P < 0.01, ^***^P < 0.001.

Through GSVA analysis, we calculated the CS-score of each AML patient in the TCGA database, and survival analysis showed that the OS of patients with high CS-score was shorter than that of patients with low CS-score (Figure 1A), indicating that the cellular senescence characteristics were correlated with the prognosis of AML patients. Pathway enrichment analysis showed that immune-related signaling pathways such as Th1 and Th2 cell differentiation, B cell receptor signaling pathway, Toll-like receptor signaling pathway, and Notch signaling pathway was significantly enriched in the high CS-score group (Figure 1B), The enrichment scores of Mismatch repair, Homologous recombination, Cell cycle, and Nucleotide excision repair were higher in the group with low CS-score (Figure 1C). Immune infiltration analysis showed that the infiltration of resting NK cells and monocytes was significant in the high CS-score group, while the proportion of resting mast cells and eosinophils was higher in the low CS-score group (Figure 1D). In addition, the expression of immune checkpoints CTLA-4, LAG3, PD-1, and CD86 was significantly up-regulated in the high CS-score group (Figure 1E).

### The expression variation landscape of CSRGs

**Figure 2:**
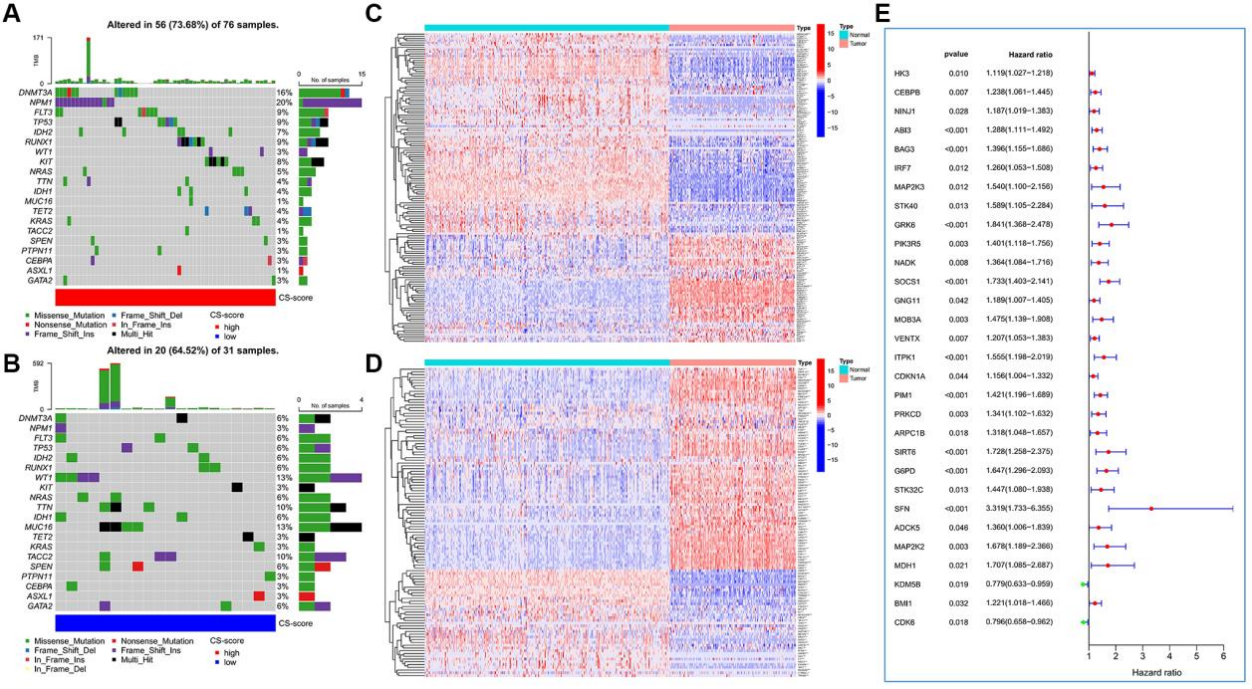
**The expression variation landscape and prognostic analysis of cellular senescence-related genes (CSRGs).** (**A**, **B**) Somatic mutation characteristics of high and low CS-score groups. (**A**) High CS-score group; (**B**) low CS-score group. (**C**) Differences in expression of cellular senescence-induced genes between AML and normal samples. (**D**) Differences in expression of cellular senescence-inhibited genes between AML and normal samples. (**E**) Identification of CSRGs significantly related to the prognosis of AML patients. ^*^P < 0.05, ^**^P < 0.01, ^***^P < 0.001.

In terms of somatic mutation characteristics, patients with high CS-score had the highest mutation frequency of DNMT3A and NPM1 (Figure 2A), while WT1, TTN, MUC16, and TACC2 were the top mutant genes in patients with low CS-score (Figure 2B). The overall CSRG expression was also significantly different in AML and normal samples. Compared with normal samples, more inhibited genes and fewer induced genes were up-regulated in AML samples (Figure 2C, 2D), indicating that AML cells had resistance to cellular senescence. Based on Cox regression analysis, we identified 30 CSRGs that were significantly associated with the prognosis of AML patients (Figure 2E).

### Construction and validation of the risk score model

**Figure 3:**
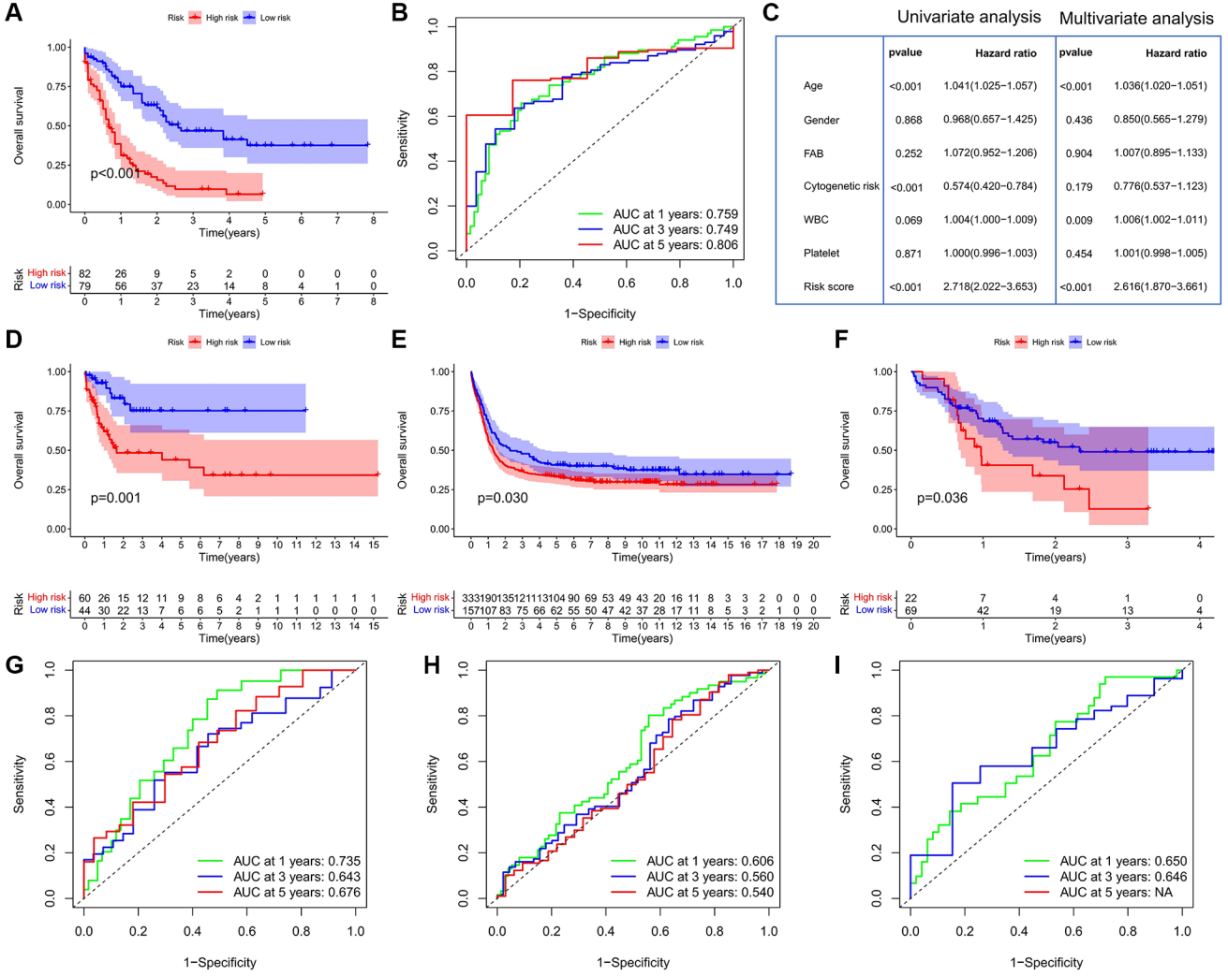
**Construction and validation of the risk score model.** (**A**) Survival analysis between the high- and low-risk score groups in the TCGA cohort. (**B**) ROC curve analysis of the risk score in the TCGA cohort. (**C**) Cox regression analysis of clinicopathologic factors and risk score in the TCGA cohort. (**D**–**F**) Survival analysis between the high- and low-risk score groups in the GEO cohorts; (**D**) GSE71014; (**E**) GSE14468; (**F**) GSE10358; Log-rank test. (**G**–**I**) ROC curve analysis of the risk score in the GEO cohorts; (**G**) GSE71014; (**H**) GSE14468; (**I**) GSE10358.

We further evaluated the prognostic value of CSRG by multivariate Cox regression analysis. Four CSRG genes (ABI3, SOCS1, PIM1, and SFN) were used to construct the risk score model. TCGA-LAML patients were divided into high- and low-risk score groups based on the optimal cut-off value. Survival analysis showed that patients in the high-risk group had significantly shorter OS than those in the low-risk group (Figure 3A). ROC curve analysis showed that the AUC values of the risk score model in predicting 1-, 3- and 5-year OS were 0.759, 0.749, and 0.806, respectively, indicating that the risk score model had high prognostic accuracy (Figure 3B). Univariate and multivariate Cox analyses showed that risk score was an independent prognostic factor for AML patients (*P* < 0.001) (Figure 3C). In all three validation cohorts, we observed that patients with high-risk scores had worse outcomes, confirming the predictive power of the risk score model (Figure 3D–3F). ROC curve analysis also confirmed the predictive accuracy of the risk score model in predicting the prognosis (Figure 3G–3I). For example, in the GSE71014 cohort, the AUC values of the risk score model for predicting 1-, 3- and 5-year OS were 0. 735, 0. 643, and 0. 676, respectively.

### Nomogram facilitate clinical decision making

**Figure 4:**
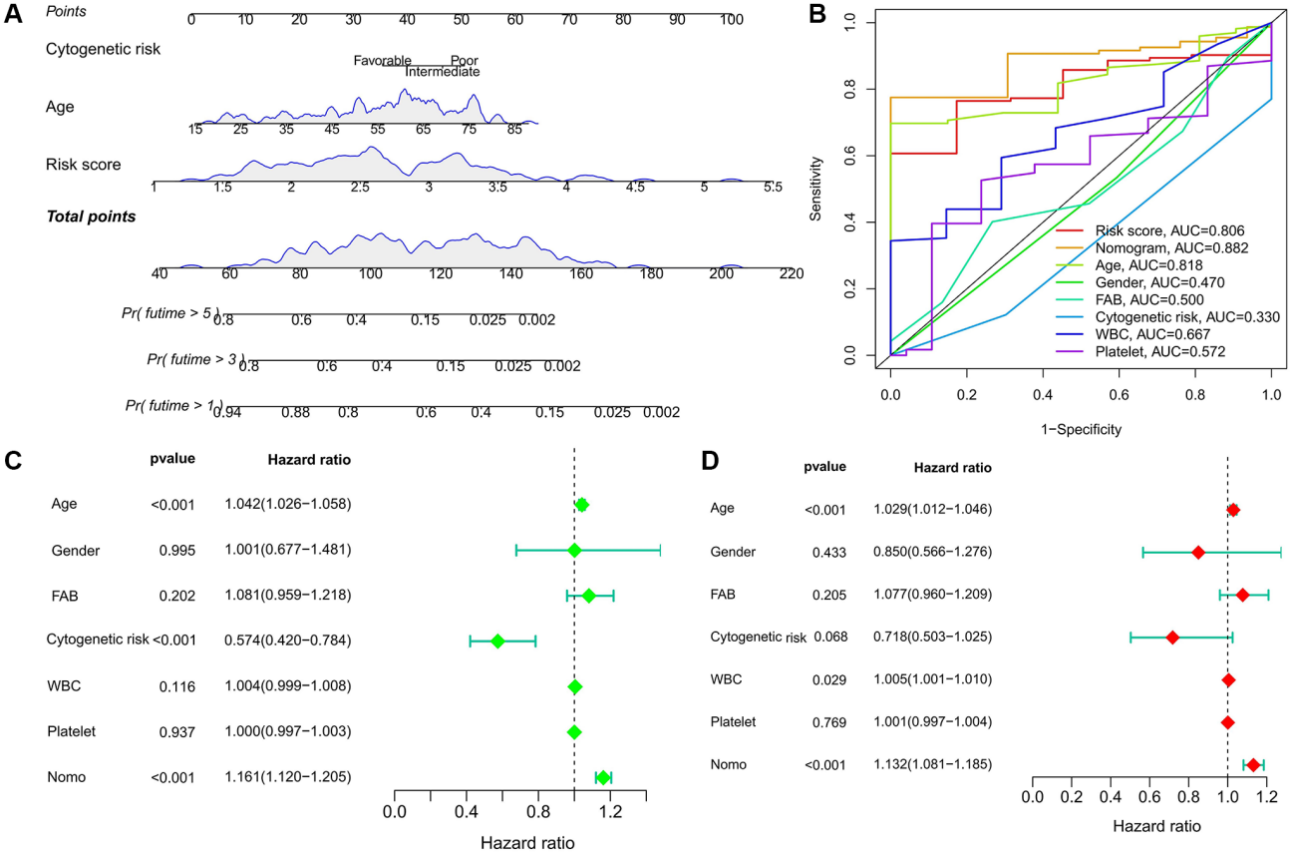
**Predictive value of risk score combined with prognosis-related clinicopathological factors.** (**A**) Nomogram predicting OS for AML patients in TCGA cohort. (**B**) ROC curves for risk score, nomogram, and other clinicopathological factors. (**C**, **D**) Cox regression analysis of the nomogram. (**C**) Univariate; (**D**) multivariate.

To better apply to clinical decision-making, we combined risk score with clinicopathological factors (cytogenetic risk and age) that were significantly associated with prognosis in AML patients to construct a nomogram (Figure 4A). Compared with other predictors, the predictive power of the nomogram was further improved (Figure 4B). Univariate and multivariate Cox analysis confirmed the independent predictive ability of the nomogram (*P* < 0.001) (Figure 4C, 4D).

### Biological characteristics and prediction of treatment response in high-and low-risk score groups

**Figure 5:**
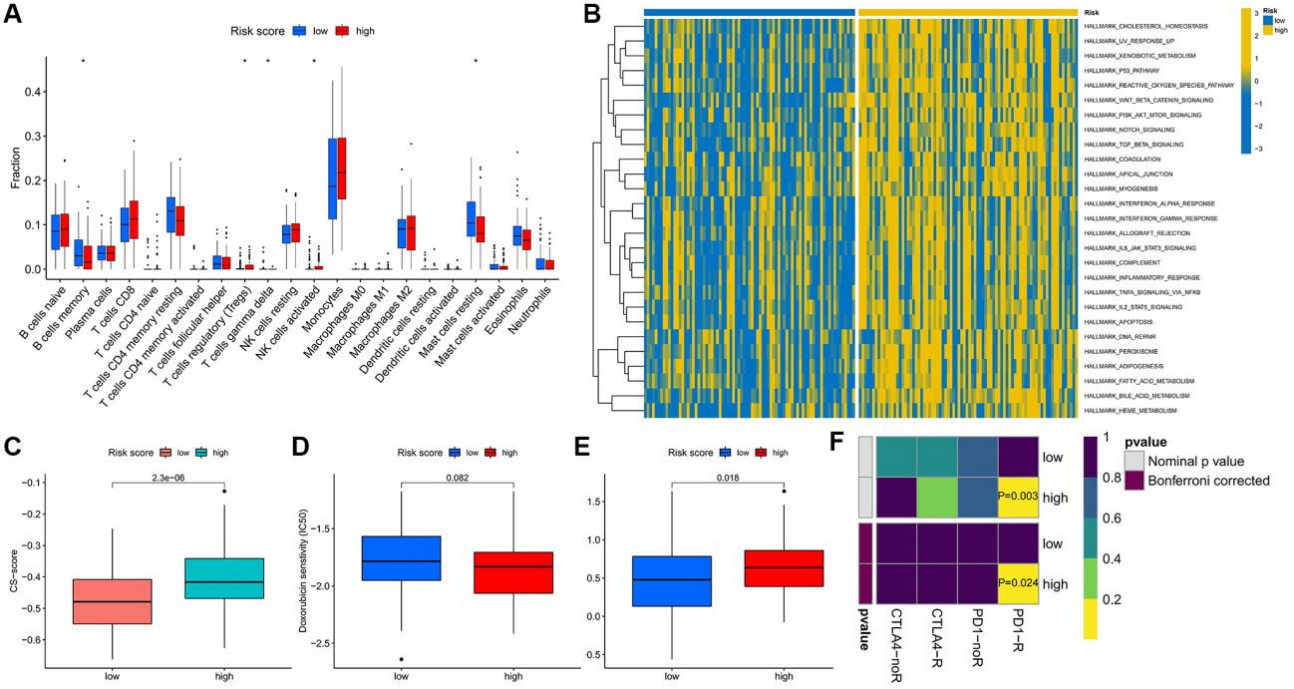
**Differences in biological characteristics and treatment response between high- and low-risk score groups.** (**A**) Infiltration levels of 22 immune cells. (**B**) Enrichment scores of cancer-related hallmark gene sets. (**C**) Differences in CS-score between high-and low-risk score groups. (**D**, **E**) Therapeutic sensitivity of two commonly used chemotherapeutic drugs for AML. (**F**) Response prediction to immunotherapy (anti-PD-1 and anti-CTLA4) between the low- and high-risk score groups. ^*^P < 0.05.

The prognosis of patients in the high- and low-risk score groups showed great differences, and we sought to explore the biological mechanisms between them. The results of immune cell infiltration analysis showed that the high-risk score group was enriched with more regulatory T cells and activated NK cells, while the low-risk score group exhibited a higher infiltrated proportion of memory B cells and resting mast cells (Figure 5A). The results of GSVA enrichment analysis then indicated the great differences between the two groups in the hallmark signaling pathways of cancer, and the activities of these pathways were all significantly higher in the high-risk score group (Figure 5B), indicating that the changes in the pathway level may be closely related to the disease development and prognosis of the two groups of patients. We also observed that the CS-score was significantly higher in the high-risk score group (Figure 5C).

In addition, we predicted sensitivity to chemotherapeutic agents and responsiveness to ICIs in both groups. Interestingly, patients in the high-risk score group were significantly less sensitive to treatment with cytarabine, more sensitive to doxorubicin (Figure 5D, 5E), and more responsive to anti-PD-1 therapy than patients in the low-risk score group (Figure 5F).

### Validation in a clinically independent cohort

**Figure 6:**
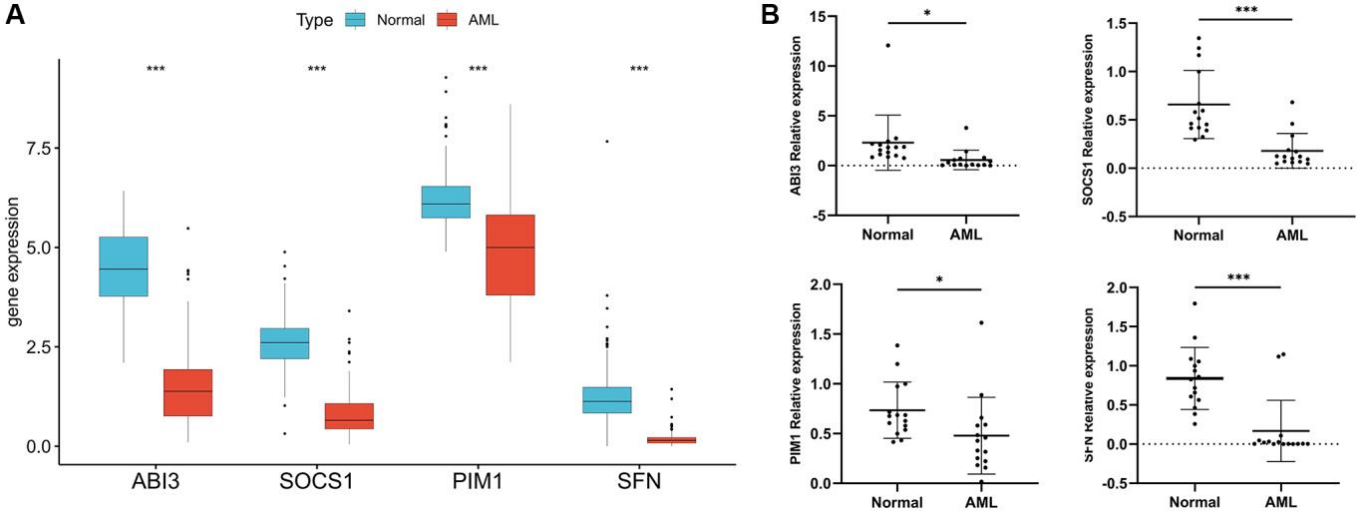
**Validation of model gene expression.** (**A**) Difference analysis of four model genes in 173 TCGA-LAML samples and 337 normal GTEx samples. (**B**) RT-PCR was used to detect the mRNA expression of the four model genes in 15 AML clinical samples and 15 normal samples. ^*^P < 0.05, ^***^P < 0.001.

TCGA cohort analysis showed that the expression of four model genes was significantly down-regulated in AML samples compared with normal samples (Figure 6A). To validate the results of bioinformatics analysis, we collected clinical samples for verification, and the results of PCR experiments also showed that the expression of several model genes was significantly down-regulated in AML samples compared with normal samples (Figure 6B). This indicates that the data analysis results are reliable and have potential research value.

## DISCUSSION

Cellular senescence is an irreversible result of cell cycle arrest [17]. It occurs not only in normal tissue cells, but also in tumor suppression under oncogene activation [18]. However, research has shown that senescent cancer cells not only inhibit tumorigenesis, but also promote tumor survival. Cellular senescence ensures the timely elimination of senescent cancer cells during the body’s immune surveillance [19]. As the relevant mechanisms continue to be clarified, inducing cellular senescence has potential anticancer properties [20].

This project aims to explore the relationship between cellular senescence and the prognosis and treatment of AML. We used CS-score to characterize the cellular senescence of AML patients by calculating the expression of cellular senescence-induced and inhibited genes. We found that patients with high CS-score had a significantly worse prognosis, accompanied by activation of immune-related signaling pathways and high expression of immune checkpoints. These findings suggest that cellular senescence in AML cells may crosstalk the immune function and mediate immunosuppression. The signaling pathway related to DNA damage repair was more active in patients with low CS-score, indicating that cellular senescence is related to the variation of genetic information. In the subsequent somatic mutation analysis, we observed that patients with high CS-score were accompanied by a higher proportion of gene mutations, while patients with low CS-score were in contrast, which may be related to their repair mechanism. In addition, we observed that the overall expression level of senescent-induced genes and inhibited genes was lower in AML samples than in normal samples, suggesting that the resistance of AML cells to cellular senescence may be one of the causes of their malignant proliferation. These results indicate that cellular senescence is closely related to the occurrence and progression of AML.

We further evaluated the prognostic value of CSRGs. By analyzing the TCGA data, we constructed the prognostic risk score signature for CSRGs. Survival time was significantly reduced in high-risk score patients, and the same results were found in the three validation cohorts. Further univariate and multivariate Cox analyses confirmed that risk score was independent prognostic factor for AML. Moreover, we combined other clinicopathological factors to construct a nomogram to predict the survival of AML patients. Encouragingly, the prognostic efficacy of the nomogram has been further improved, which has important reference value for clinical decision-making. Cytarabine and anthracycline drugs such as doxorubicin are commonly used chemotherapeutics in the clinical treatment of AML. We predicted the sensitivity of patients with high and low risk scores to these drugs. Patients with high-risk scores showed significantly reduced sensitivity to cytarabine and showed higher sensitivity to doxorubicin. We also observed that high-risk score patients had a higher CS-score, suggesting that high-risk score patients may benefit more from treatment with ICIs. Interestingly, high-risk score patients had a higher predicted response to immunotherapy with anti-PD-1 compared to low-risk score patients. Related studies have shown that the enhancement of senescence-related secretory phenotype of tumor cells can increase the sensitivity to ICIs [21]. The loss of senescent-induced genes and the amplification of inhibited genes are one of intrinsic reasons for tumor cells to resist ICIs [22].

Finally, we verified the expression of the four model genes in AML samples by RT-PCR, and the experimental results were consistent with the bioinformatics results, indicating the reliability of our analysis. Among the four model genes, SOCS1 is a widely recognized tumor suppressor gene, and its downregulation has been detected in various malignant tumors, including AML [23]. In AML cells, Gfi-1 can silence SOCS1 through epigenetic modification [24]. Moreover, the silencing of SOCS1 by gene methylation overcomes the inhibition of SOCS2 on the downstream JAK1/STAT signaling pathway and promotes the growth and proliferation of AML cells [25]. PIM1 protein is the product of proto-oncogenes family [26]. As a serine-threonine kinase, its expression is up-regulated in various malignant tumors and plays a significant role in promoting cancer by enhancing hematopoietic cell survival and inhibiting cell apoptosis [27, 28]. PIM1 expression appears to be upregulated by STAT5 and is overexpressed in primary AML blast samples [29]. Notably, PIM1 has been implicated in FLT3-mediated leukemogenesis in FLT3-ITD AML and may be an effective therapeutic target for this disease [30, 31]. Up-regulation of SFN expression is associated with cisplatin chemotherapy failure and poor prognosis in NSCLC [32], and it regulates lung cancer progression through the induction of autophagy by nucleating Vps34-BECN1-TRAF6 complex [33]. SFN may also interact with CDC25B to promote the growth and proliferation of HCC [34]. Previous reports have shown that ABI3 expression is lost in follicular thyroid carcinoma and its restoration significantly inhibits cell proliferation, invasion, migration and tumor formation, acting as a tumor suppressor gene [35, 36]. However, few studies on SFN and ABI3 in AML have been reported, and further studies are needed to explore their biological functions in AML.

In conclusion, the risk score model constructed based on CSRGs can be used to evaluate the prognosis of AML patients and guide clinical treatment. Our study not only provides new predictive signals for the prognosis of AML, but also guides the future treatment of AML. However, our study also has certain limitations. The prognostic independence and accuracy of the risk score model and nomogram need to be validated in more AML cohorts with clinical data, and the biological function of the risk model genes in AML cells remains to be elucidated. We will conduct these studies in future projects to fully reveal the biological value of CSRGs in AML.
